# The Story of the Insane from Year to Year

**Published:** 1889-08-17

**Authors:** 


### The Story of the Insane from Year to Year.

Asylum Reports.

### DORSET COUNTY ASYLUMS.

There are two asylums situated within a mile or two of each other, and both are under the same management. In the year 1848 there were but 147 patients, and now there are 426. Since the opening of the asylum, the average annual percentage of recoveries has been 35·9. Last year it was 41·9, excluding transfers. The average annual death-rate has been 7·1, while last year it rose to 9·5, but even that is under the average of asylums. The number of patients at the end of 1888 was exactly the same as that of 1887—a state of things very unusual in county asylums. We learn from the report that the attendants and nurses have been provided with uniforms for the first time. Seventy-six patients were admitted during the year; 41 died, and 27 were discharged. Ten of the admissions were owing to hereditary tendency, and seven to alcoholism. Dr. Macdonald says that 83 per cent. of the male cases were incurable on admission, and he rightly thinks that these cases could not be properly cared for at home. He says: "To send these cases to the workhouse, where no special means of treatment is provided, does not commend itself to those who know the trouble and trial such cases often are. In the interests of these too often mental and physical wrecks of humanity, I hope no mistaken or erroneous notion of how they might be treated will prevent their early removal to the asylum, where, if not cured—for they cannot be renewed—they are at any rate nursed and taken care of." Speaking of idiot children, Dr. Macdonald bewails their presence in the general wards, and thinks Dorset and the adjoining counties might join in providing an asylum for idiots. So they *might*; but Dr. Macdonald will find the years slip by, and his old age advancing, and his energy waning, and the asylum still unbuilt.

### SURREY COUNTY ASYLUM AT CANE HILL.

It is five years since this asylum was opened, and it contained 999 patients at the expiration of the first complete year of its existence. There are now 1,072 in the asylum. It is proposed to enlarge the building so that it will contain 2,000 beds, a step which we should say is of more than doubtful expediency. No asylum should ever contain more than 1,000 patients. The recoveries stand at 40·12 per cent. on the admissions, excluding transfers, and the death-rate is 10·62 per cent. on the average number resident. Not fewer than 78 of the admissions were owing to hereditary tendency, and 63 to alcoholic excess. Phthisis was the cause of death in 13 cases. The weekly rate of maintenance is nearly 9s. 8d., which is considerably above the average. Dr. Moody says, when referring to Dr. Hill's appointment to Wandsworth: "The fact of my two first assistants having been appointed to superintend important asylums, within five years of the opening of this institution, is a source of gratification to me, and is an indication of the good reputation Cane Hill has established for itself."

### MONTROSE ROYAL ASYLUM.

We have often wondered, and have expressed our surprise, that England has not adopted this form of asylum constitution which really includes all that is good in both public and private institutions for the insane. The Montrose Asylum contained at the close of their year (May 15, 1889) 504 patients. The admissions number 113, and 23 of these were private patients; 73 were discharged, and 35 died. The percentage of recoveries calculated on the admissions is 40·7. The death-rate was 7· per cent. on the average number resident. Restraint was used on two occasions. Referring to the question of the hospital treatment of the insane, Dr. Howden has the following sensible remarks: "Proposals have been made lately in the London County Council to establish hospitals for the treatment of cases of insanity, where the highest medical knowledge and skill will be brought to bear on the study and treatment of insanity. If this project be carried out, it will no doubt be an advantage to the medical schools, by affording extended means for the study of mental diseases; but that it will be a benefit to the patients is, I think, more than doubtful, as I do not consider than any asylum can efficiently fulfil its curative functions unless it has plenty of land around it, to afford scope for the occupation and exercise of the patients." It may be questioned whether the means available at present for clinical instruction in insanity have been thoroughly taken advantage of; and Dr. Howden might have gone further and said that the treatment of the insane in a hospital in a crowded city is in many cases likely to be injurious, and delay or prevent recovery.

### ROYAL ALBERT ASYLUM, LANCASTER.

This institution is for idiots and imbeciles only. It is not a county asylum, but pauper idiots are admitted to a certain extent from some of the northern counties at rates of payment similar to those of the county asylum to which the patients would, in the ordinary course, have been sent, and there are many cases paid for by their friends. Some of the private patients are accommodated in a handsome villa, not far from the main building. Including private cases, there were 553 in residence at the end of the year; 89 new cases were admitted, 64 were discharged, and 24 died. One patient was discharged recovered, 19 were discharged much improved, and 38 either moderately or slightly improved. Dr. Shuttleworth's report is a very able one. He travels carefully over the whole ground, and presents us with an account of the institution, from which we can form an accurate idea of the good work it has done during the past year. The mortality was only 4·4, but even this low rate is above the average of recent years.

### KILLARNEY DISTRICT ASYLUM.

There were 390 patients in this asylum on the 31st December, 1888. The admissions were 91, the discharges 54, and the deaths 33. The recoveries reached the creditable percentage of 48·3, and the death-rate was 8·4 per cent., adopting the usual method in both cases. The weekly maintenance rate is about 8s. 4d. In five of the admissions the disease was cansed by intemperance, and in 19 it was hereditary. Dr. Woods specially mentions the following cases when referring to the admissions: "Amongst the admissions were five members of one family—mother, son, and three daughters—all acutely maniacal, the insanity in at least four of the cases, not having existed more than three days." Additions are being made to the female wing of the asylum.

### KIRKLANDS ASYLUM, NEAR GLASGOW.

This asylum admits patients from the lunacy districts of Govan, Lanark, and the city of Glasgow. During the year 78 cases were admitted, 65 were discharged, and 12 died. The percentages reckoned in the usual way show that 48·7 recovered, and 5·4 died. The recoveries are considerably above the average, and the deaths considerably under it. We gladly endorse Dr. Clark's remarks on the nursing staff. He says: "The whole question of the life of attendants and nurses in our asylums is a large one, and is only being faced bit by bit year by year. Their quarters, hours on duty, and recreations are now becoming questions of practical politics in asylum work, and better conditions are likely to be secured for them in the future than they have experienced in the past." We did not notice any table giving the weekly cost of maintenance per head.

